# The impact of non-caloric artificial sweetener aspartame on female reproductive system in mice model

**DOI:** 10.1186/s12958-023-01115-4

**Published:** 2023-08-14

**Authors:** Ab Qayoom Naik, Tabassum Zafar, Vinoy K Shrivastava

**Affiliations:** 1 Department of Zoology, Govt. Degree College, Paloura Mishriwala, Jammu, J & K 180018 India; 2 Laboratory of Endocrinology, Department of Biosciences, Barkatullah University, Bhopal, M. P 462026 India

**Keywords:** Aspartame, Artificial sweeteners, Reproductive toxicity, Female infertility

## Abstract

**Background:**

Artificial sweeteners, used as sugar substitutes have found their ways into almost all the food items due to the notion that they are non-caloric. Aspartame is used in numerous food products throughout the world. The primary users of aspartame include diabetics and calorie conscious people who intend to limit their calorie intake.

**Methods:**

Female Swiss albino mice were divided into three groups (12 mice each) for the duration of 30 and 60 days consecutively. The treatment groups received 40 mg/kg b. w. aspartame orally. Hormone assays using ELISA and tissue histopathology have been performed along with the fertility assay to access the treatment outcomeon the fertility of treated mice in comparison to controls.

**Results:**

Present study reports that female mice treated with aspartame for 30 and 60 days showed significant reduction in body weight, relative organ weight of (liver and kidney) and gonadosomatic index. These changes were more significantly recorded in 60 days treatment group. Aspartame treated animals for 30 and 60 days showed duration-dependent decrease gonandotropins (follicle stimulating hormone and luteinizing hormone), and steroids (estradiol and progesterone). Moreover, severe histopathological changes, reduction in number of growing follicles, degenerative changes in follicular structure, corona radiata and zonagranulosa were also observed. Besides, histomorphological changes were also observed in the uterine structure including atrophic uterine endometrial glands, contracted endometrial lining, disruption of the endometrial structure and the shapes of blood vessels were also altered.

**Conclusion:**

Non-nutritive artificial sweeteners including aspartame negatively impact the function of ovaries and feedback mechanism of reproductive hormones by affecting the hypothalamic–pituitary–gonadal axis. In light of present findings the aspartame negatively impacted the reproductive system of female mice. More studies are required to identify the molecular mechanism and the pathways involved.

## Background

Humans and other animals naturally like sweet tastes, which boosts eating satisfaction [1, 2]. Sugar is bad for teeth, waistline, and is associated with several degenerative disorders. Sugar intake may affect behaviour, emotions, health, and disease [3–5]. The number of people suffering from diabetes [6], obesity [7], hypertension [8], and heart disease [9] is increasing every year. Increased sugar in foods, desserts, and drinks has prompted health concerns. Added sugar intake has increased worldwide obesity [10]. The World Health Organization recommends that added sugars should make up no more than 10% of daily caloric intake for optimal health [11]. Likewise, American Heart Association (AHA) recommendation is 6 table spoon full (24 g, providing 100 calories) of sugar per day for women and 9 table spoon full (36 g, providing 150 calories) of sugar per day for men [12].

The popularity of sugar free foods is attributed to their low calorie content. A number of low calorie artificial sweeteners acesulfame K, aspartame, neotame, saccharin, stevia, and sucralose [13, 14] having calorie value lower than sugar are produced throughout the world in a very large quantity. The admissible daily intake (ADI) for aspartame is 40 mg per kilogram body weight (for humans) as stated by FDA. However, this value has given birth to a number of controversies, wherein, many researchers have reported that not only the ADI but lesser quantities are not totally safe for consumption [15, 16]. A number of health ailments like metabolic syndrome, increased weight gain and other negative health effects have been associated with sugar intake [17–20].This has finally resulted into the promotion of ASs as a healthy alternative [13, 21]. Sugar substitute is an artificial non-nutritive sweetener which mimics the effect of sugar on taste [22]. Artificial sweeteners (ASs) are also known as non-nutritive or intense sweeteners because the sweetening potential of ASs is very high compared to common sugar. Aspartame containing foods provide a kind of dietary option which is considered helpful in containing obesity or diabetes mellitus [23].

However, ASs has been associated with numerous adverse effects [24, 25]. Many studies have investigated the impact of ASs exposure during pregnancy and early childhood but due to the obscure conclusions of the studies about the impact of the ASs during critical developmental periods adds to the controversy [26–28]. According to the American Dietetic Association ASs consumption is safe in children and pregnant women within acceptable intake limits [13], however, the US Institute of Medicine states a paucity of evidence of ASs safety and suggests avoiding ASs use in childhood [29]. Food and beverage intake of non-nutritive sweeteners (NNSs) has increased worldwide over the last three decades. Consumers’ preference of NNSs rather than sugar or other healthy sweeteners might be due to their ability to minimize weight gain [30]. ASs are so abundant and widespread in the food industry that a number of people even do not know that they are consuming them [31]. On the basis of some studies supporting the use of ASs, if their use for human consumption is deemed safe [32, 33], significant evidences suggest that ASs may not be necessarily healthy, do not mitigate weight gain and may not be good to improve circulating glucose levels [34].

Gut-brain axis plays a vital role in sensing of foods ingested by humans. Feedback circuits are initiated by this axis to modify gene expression and regulate glycemia, satiety, and energy partitioning [35, 36]. Aspartame (ASP) is one of the most commonly used ASs and is used as sugar substitute in a number of food products including, soft drinks, jams, chewing gum, canned fruit, candies, cosmetic products, vitamins, and medications [37, 38]. There are hundreds of millions of aspartame consumers throughout the world. Children and women of child bearing age are the major users of ASP [39, 40]. The consequences of ASP intake in pregnant women have been minimally addressed. A few studies investigating the impact of ASP on the gestation in humans are available. Besides, a few studies, in which the effect of ASP on the weight and physiology of offspring have been studied, are also available [41, 42].

## Materials and methods

### Animal model and aspartame administration

The animals used in this study were maintained in the animal centre of the Department of Biosciences in accordance with the Institutional Animal Ethical Committee (IAEC) and Committee for Control and Supervision of Experiments on Animals(CCSEA), New Delhi, India, (No. 1885/GO/S/16/CPCSEA/IAEC//B.U./08 Dt. 18/06/16). The female mice were housed in standard polypropylene mice cages (290 × 220 × 140 mm) containing rice husk as bedding material. The animal room was well ventilated and maintained under standard experimental conditions (Temperature 22 ± 2ºC and 12 h light/dark cycle) throughout the experimental period. All the animals were provided with standard pellet diet and water *ad libitum*. The animals were acclimatized to the standard laboratory conditions for one week prior to experimental use. Healthy female albino mice Parkes (P) strain (5 to 7 weeks old) of 20 ± 2 g body weight was used in this study. Twelve mice were kept in the each group among, six were used for fertility assay and six were sacrificed for serum and tissue analysis for each duration. The animals were purchased from the College of Veterinary Sciences, Mahow, Indore, Madhya Pradesh, India. The animals were randomly assigned to each experimental group. Aspartame (C_14_H_18_N_2_O_5_, 99% pure CDH- Laboratory Chemicals India) was purchased in powder form and mixed in water to make it suitable for the oral ingestion. The control groups received distilled water by oral gavage and aspartame groups received 40 mg/kg b. w./day aspartame (2 mg/ml/2000 ppm) dissolved in distilled water for 30 and 60 days, until the completion of the study. The dose and duration of aspartame (ASP) used in the present study were based upon the previous studies [42–44]. At the end of the experiment, six female mice from each group were used for analysis or relative organ weights (liver and kidney), GSI, hormonal analysis and histopathological examinations of ovaries and uteri and another six animals were used for fertility studies.

### Fertility assessment

After the aspartame treatment for 30 and 60 days the females were housed with virgin untreated males in the ratio of 2:1. The males were removed after 24 h and the successful mating was confirmed by the presence of vaginal plug. The females were observed for successful pregnancy, fertility rate, gestational length, litter size, litters weight [45].

### Serum sampling and processing

At the end of 30 and 60 days aspartame administration, the female mice were sacrificed and blood samples were collected by cardiac puncture and centrifuged at 3000 rpm for 10 min to obtain serum. The serum samples were further used for the analyses of luteinizing hormone(LH), estradiol (E2), progesterone(P4), and follicle-stimulating hormone(FSH) levels.

### Body weight, relative organs weight, and gonadosomatic indices (GSI)

The body weights of the experimental animals, control as well as treatment were recorded at the initial day i.e. zero days and at the end of the different durations of the experiment i.e. 30 and 60 days. The values were expressed in grams [46].

The body weight gain was calculated as:

*Body weight gain = Final body weight-Initial body weight*.

Whereas, relative organs weight (liver and kidney) was calculated as:

*Relative organs weight = weight of organs (g) ÷ Final weight x 100*.

GSI were calculated by the following formula [47].

*GSI = Gonad (ovary) weight (mg) ÷ Body weight (g) x 100*.

### Estimation of Follicle Stimulating Hormone (FSH), Luteinizing Hormone (LH), Progesterone (P4), and Estrogen (E2) in the serum of female mice

The levels of E2, P4, FSH and LH were detected using mouse specific enzyme-linked immunosorbent assay (ELISA Kits) (Calbiotech Inc CA United States), according to the manufacturer’s recommended instructions.

### Histopathology

Animals of the treatment as well as control groups were sacrificed at the end of the experiment i.e. 30 and 60 days duration. Liver, kidneys, ovaries and uterus were dissected out immediately after the animals were sacrificed, washed in cold 0.9% NaCl, cleared of any attached tissues and fats, and were blotted dry. Small sections of all the organs were placed into separate vials containing Bouin’s fixative. All the vials were labelled with name of the organ, date of fixation and group details. 5 µ thick paraffin embedded tissue sections were cut, and stained with Ehrlich’s Hematoxylin and Eosin [48]. The stained sections were observed under compound microscope at 100X and 400X magnifications for any histopathological changes. The microphotograps of the observed tissue sections were taken by using microphotography unit.

### Statistical analysis

The observations and data were tested for statistical significance using SPSS software. The values were expressed as mean standard deviation (Mean ± SD). One way ANOVA is performed to analyse the statistical differences among groups.

p < 0.05 is considered significant.

## Results

### Body weight

The animals were weighed initially and after different intervals i.e. 30 and 60 days of the aspartame experiment. The animals treated orally with ASP showed a significant (p ≤ 0.01) decrease in their body weight when compared to the respective controls. The decrease in body weight was significant (p ≤ 0.001) in duration dependent manner (Table 1).

**Table 1:** Body weight (g), relative organ weight (g/100 g b. w.), and GSI (mg/100 g b. w.) of control and aspartame (ASP) treated female mice after 30 and 60 days

**Groups**	**Initial body weight**		**Final body weight**		**Percent weight gain**	
Control	20.00 ± 2.00		25.50 ± 1.05		27.5%	
ASP-30	20.33 ± 2.06		19.83 ± 1.17^**^		-2.46%	
ASP-60	20.17 ± 2.79		21.33 ± 1.21^**^		5.75%	
**Relative organ weight (g/100 g b. w.)**		**Duration** **(days of treatment)**		**Groups**		
				**Control**		**ASP**
Liver		30		4.77 ± 0.12		3.76 ± 0.28**
		60		4.95 ± 0.69		3.12 ± 0.04**
Kidney		30		0.59 ± 0.03		0.39 ± 0.04**
		60		0.60 ± 0.03		0.36 ± 0.05**
**Gonadosomatic indices (GSI)**		**Duration** **(days of treatment)**		**Groups**		
				**Control**		**ASP**
		30		39.3 ± 3.22		26.69 ± 2.86^**^
		60		48.33 ± 5.66		26.35 ± 4.93^**^

± SD of six animals

*Significant difference (p ≤ 0.05) compared to control by one way ANOVA

**More significant difference (p ≤ 0.01) compared to control by one way ANOVA

***Highly significant difference (p ≤ 0.001) compared to control by one way ANOVA

^NS^ Non significant compared to control by one way ANOVA

### Relative organs weight and gonadosomatic indices

After treatment of ASP for 30 and 60 days, the relative organ weights of liver and kidney were decreased significantly (p ≤ 0.01) in ASP treated group when compared to control groups. Decrease in relative organ weights of liver and kidney was highly significant (p ≤ 0.001) in 60 days ASP treated group. There were no significant changes in relative organ weights in control groups. The ovarian weights reduced significantly (p ≤ 0.01) after the treatment of ASP for 30 and 60 and days, as compared to control groups. The reduction in ovarian and uterus weight was significant (p ≤ 0.001) in 60 days of ASP treated group (Table 1).

### Hormone analysis

**Fig. 1:**
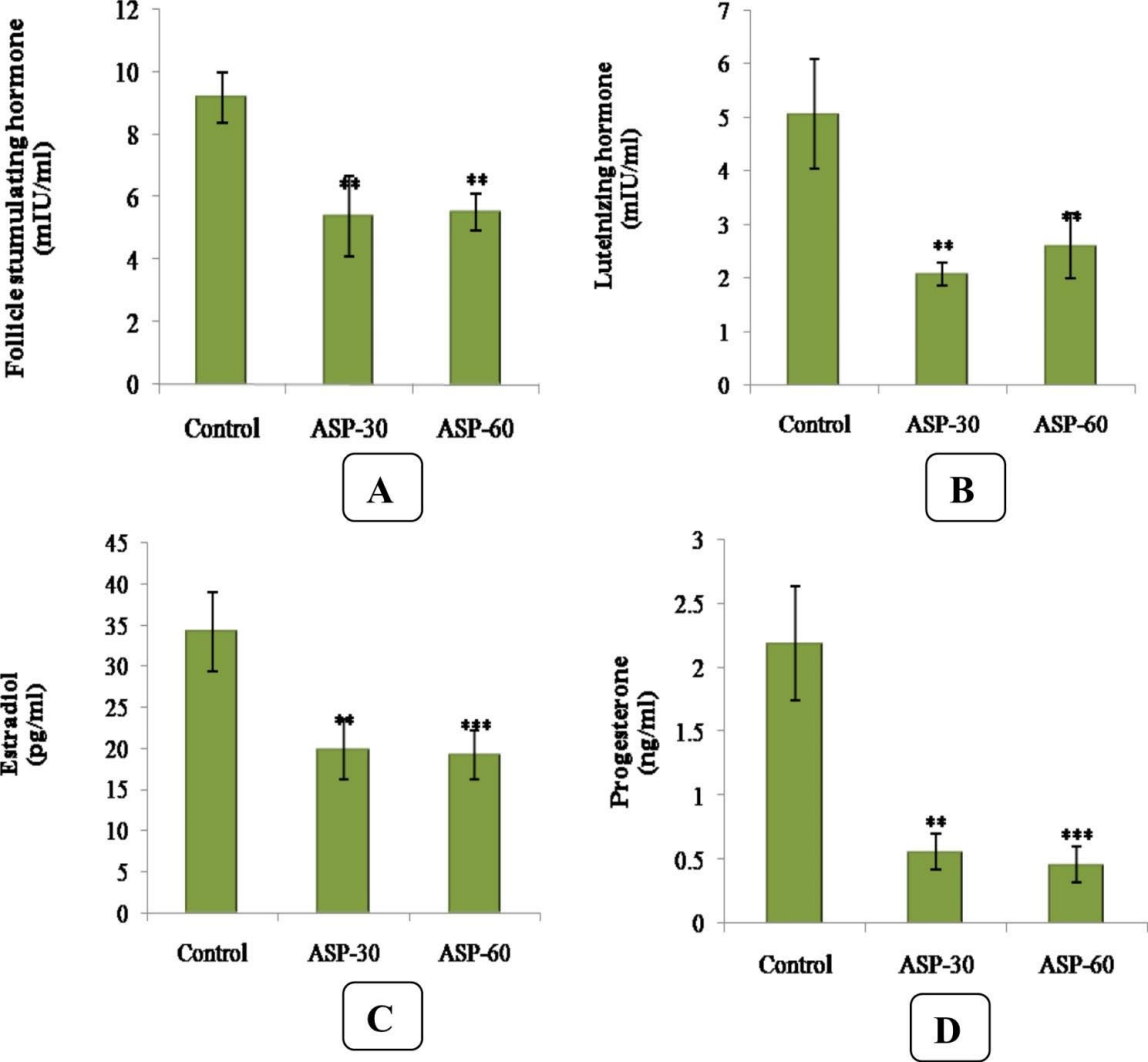
**(A)**. Follicle stimulating hormone (FSH) level (mIU/mL) of Aspartame (ASP) after 30 and 60 days compared to Control. **(B)**. Luteinizing Hormone (LH) level (mIU/mL) of Aspartame (ASP) treated female mice after 30 and 60 days compared to Control. **(C)**. Progesterone (P4) level (ng/mL) of Aspartame (ASP) treated female mice after 30 and 60 days compared to Control. **(D)**. Estrogen (E2) level (pg/mL) of Aspartame (ASP) treated female mice after 30 and 60 days compared to Control

Animals treated with aspartame for 30 and 60 days showed a significant (p < 0.01) decrease in FSH and LH hormonal levels as compared to control group. The decrease was significant (p < 0.001) in 60 days treated group. In addition, estradiol (E2) and progesterone (P4) levels also showed a significant (p < 0.01) decrease in 30 and 60 days of ASP treated groups when compared to control groups (Fig. 1).

### Follicle count and fertility parameter

Females treated with ASP showed significant (p ≤ 0.05) duration-dependent decrease in follicle count compared to control including primordial and growing follicles. Besides, animals treated with ASP showed duration dependent decrease in litter size, body weight of pups postnatal viability, weaning index and fertility index compared to control group. However, no significant difference was observed in gestation period in 30 and 60 days ASP treated groups compared to control (Tables 2 and 3).

**Table 2:** Follicular count (Ovarian reserves) of Control and Aspartame treated mice after 30 and 60 days

Follicle Count	Groups	Duration	
		30 days	60 days
Primordial follicles	Control	15.8 ± 2.59	16 ± 2.24
	ASP	11.8 ± 1.92^*^	8.4 ± 1.14^**^
Primary follicles	Control	4 ± 1.58	4.4 ± 1.14
	ASP	2 ± 1.0^*^	1.4 ± 0.55^**^
Secondary follicles	Control	5.2 ± 1.79	5.4 ± 1.82
	ASP	2.4 ± 1.14^**^	1.8 ± 0.84^**^
Graffian follicles	Control	1.8 ± 0.84	2.2 ± 0.84
	ASP	0.6 ± 0.55^*^	0.4 ± 0.55^**^
Atretic follicles	Control	1.4 ± 0.55	1.6 ± 0.55
	ASP	5.2 ± 1.48^*^	9.4 ± 2.7^**^

± SD of six animals

*Significant difference (p ≤ 0.05) compared to control by one way ANOVA

**More significant difference (p ≤ 0.01) compared to control by one way ANOVA

***Highly significant difference (p ≤ 0.001) compared to control by one way ANOVA

^NS^ Non significant compared to control by one way ANOVA

**Table 3:** Reproductive indices of Control and Aspartame (ASP) treated female mice after 30 and 60 days

Parameters	Group	Duration (days)	
		30 days	60 days
Fertility index (FI)	Control	100%	100%
	ASP	80%	60%
Gestation period	Control	20.6 ± 1.14	20.8 ± 0.84
	ASP	19.2 ± 0.84 ^NS^	19.4 ± 0.55 ^NS^
Litter size	Control	7.4 ± 0.55	7.6 ± 0.55
	ASP	5.8 ± 0.45*	5.4 ± 0.89^**^
Litter weight (g)	Control	1.55 ± 0.09	1.57 ± 0.08
	ASP	1.40 ± 0.04^*^	1.34 ± 0.07^**^
Postnatal viability index	Control	100%	100%
	ASP	96.55%	96.29%
Weaning index	Control	97.29%	100%
	ASP	93.10%	92.59%

± SD of six animals

*Significant difference (p ≤ 0.05) compared to control by one way ANOVA

**More significant difference (p ≤ 0.01) compared to control by one way ANOVA

***Highly significant difference (p ≤ 0.001) compared to control by one way ANOVA

^NS^ Non significant compared to control by one way ANOVA

### Histopathological and histomorphological analysis

**Fig. 2:**
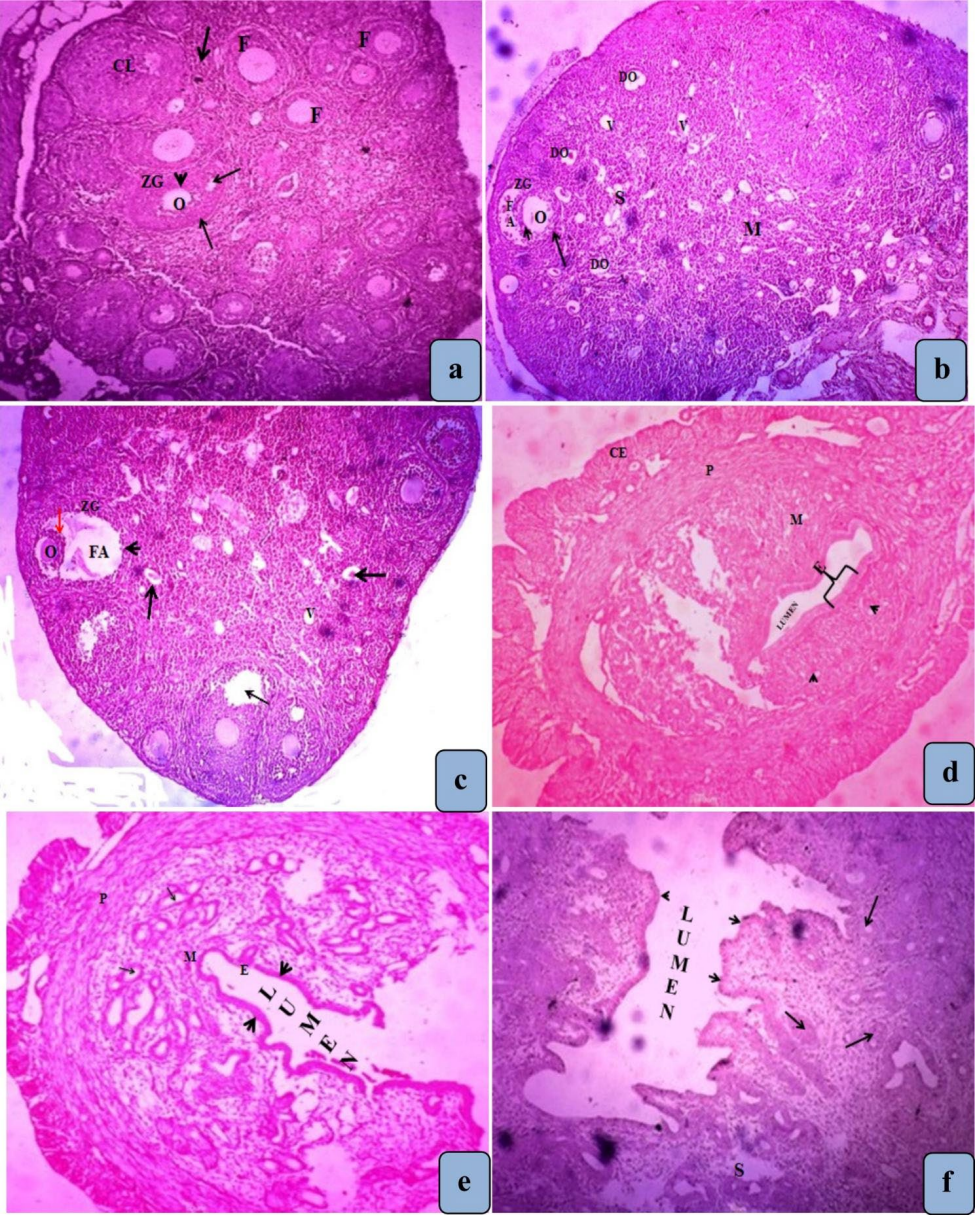
**(a)** Section of ovary of control mice showing normal growing follicles (F) of different sizes and stages. **(b)** Section of ovary of mice treated with ASP for 30 days showing decreased number of growing follicles. Degenerative changes were observed in follicular structure like degenerating oocytes (DO), theca follicli (arrow), corona radiata (arrow head) and Zona granulosa (ZG). **(c)** Aspartame treated ovary for 60 days shows degeneration of follicular antrum (FA), corona radiate (red arrow), oocyte (O), zona granulose (ZG) and theca follicle (arrow head). The number of follicles is very less. **(d)** of control uterus showing perimetrium (P), myommetrium (M), and endometrium (E). **(e)** Section of uterus of mice treated with ASP for 30 days shows compressed endometrial cells (arrow heads) and the glands (arrows) are also atrophic, attributed to endometrium destruction due to ASP. **(f)** Uterus treated with ASP for 60 days shows degeneration in uterine endometrial lining (arrow head). Endometrial glands were also affected as their number was decreased and their epithelial lining showed necrosis (arrows) **(H & E × 100)**

**Fig. 3:**
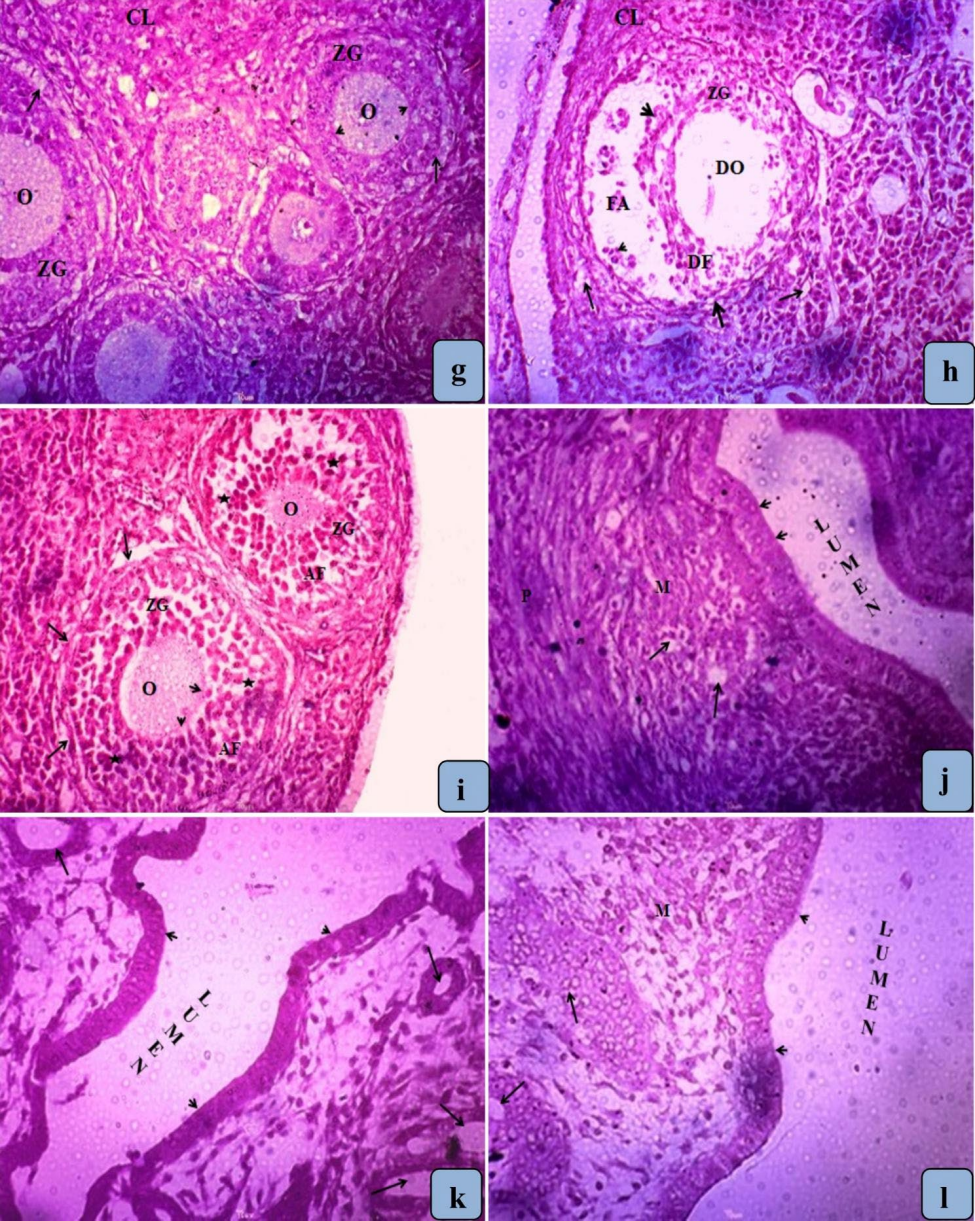
**(g)** Magnified view of control ovary section showing developing follicles with normal histological features characterized by well defined granulose cells (GC) surrounding the oocyte. **(h)** Section of ovary treated with ASP for 30 days shows degenerative changes in follicles (DF), degeneration of follicular antrium (FA), primary Oocyte (O), theca follicli (thick arrow), corona radiata (arrow head) and zona granulosa (ZG) and decreased number of primary follicles. **(i)** Section of ovary treated with ASP for 60 days shows degenerative changes in thecal layer of follicle membrane (arrows), zona granulose (ZG), and zona pellucida (arrow head). The appearance of pyknotic bodies (atretic bodies) in granulosa cells (star) and granulation of cytoplasm are the indications of early follicular atresia. **(j)** Uterus of control mice showing normal endometrium with *stratum basale* and *stratum functionalis* bearing fibrous connective tissue and normal tubular glands (arrows). **(k)** Uterus treated with ASP for 30 days shows atrophic uterine endometrial glands (arrows), squeezed endometrial lining (arrow head) and the shapes of blood vessels were also altered (arrows). **(l).** Uterus treated with ASP for 60 days shows larger endometrial cells (arrow head) and atrophic endometrial glands (arrows). Myometrium (M) also appears atrophic due to atrophy of the smooth muscle cells **(H & E × 400)**

The ovary female mice of the control group showed normal histoarchitecture including normal follicles, different sizes and stages of developing oocyte, zona granulose and thecal layers of follicle and corpus luteum (Figs. 2a and 3a). However, the female mice exposed with ASP for 30 (Figs. 2b and 3b) and 60 days (Figs. 2c and 3c) showed histopathological changes in the ovarian structure characterized by decreased number of growing follicles, degenerative changes were observed in follicular structure i.e. degenerating oocytes, theca layers, corona radiata and zonagranulosa. The connective tissue of medullary region also showed some degenerative changes and vacuolization. .

Female mice of control group presented a normal uterus histoarchitecture with normal endometrium, endometrial glands, myometrium, intact perimetrium and simple columnar epithelium (Figs. 2d and 3d). Animals treated with aspartame for 30 days (Figs. 2e and 3e) and 60 days (Figs. 2f and 3f) presented histomorphological changes in the uterine structure including atrophic uterine endometrial glands, squeezed endometrial lining, disruption of the endometrium and the shapes of blood vessels were also altered.

## Discussion

The extensive use of artificial sweeteners particularly aspartame and its associated negative effects have been investigated by many researchers. However, reproductive toxicity of aspartame has been minimally addressed.

The results of the present study showed that ASP administration induced duration dependent decrease in body weight. The reduction in body weight was significant (p < 0.001) in 60 days treatment group as compared to the control. The reduction in body weight may be due to reduced food and water intake which may have been caused by ASP consumption. These observations are supported by some researchers [49, 50] who reported that ASP intake induces satiety, decreases food intake and bodyweight. ASP administration increases circulating blood levels of phenylalanine which is reported to suppress food intake in humans and animals and increases cholecystokinin secretion which delays the gastric emptying [50–53]. ASP was also reported to reduce body weight and fat mass in overweight subjects [50, 54–56]. Some researchers believe that decreased body weight in ASP treated animals is a result of diminution of Neuropeptide Y (NPY) in its principal hypothalamic site of synthesis [57]. Neuropeptide Y (NPY) inhibits lipolysis and stimulates de novo lipogenesis and thus promotes weight gain and fat deposition [58–61].

The relative organ weight and GSI of animals treated with ASP significantly (p < 0.01) decreased after 30 and 60 days of ASP administration. The observed decrease in relative organ weight and GSI were significant (p < 0.01) in 60 days ASP treated group as compared to the control group. The measurement of relative organ weight and GSI is an important indicator to study the organ toxicity due to the exposure of any toxic chemical. The duration-dependent decrease in relative organ weight and GSI suggest abnormalities and atrophy in these organs. The results observed suggest that ASP may have toxic effects on liver, kidney and ovary which may have been caused by the methanol intoxication that increase the lipid peroxidation (LPO) [62, 63]. It was reported that aspartame can act as chemical stressor by increasing corticosteroid level which in turn has been shown to decrease the size and weight of organs due to oxidative damage [64, 65]. Formaldehyde, which is the first metabolic product of methanol, increases the population of shrunken and dead cells [66–68] which might be responsible for decreased organ weight.

Aspartame treated animals for 30 and 60 days showed duration-dependent decrease in androgens (FSH and LH), and steroid (E2 and P4) hormone levels. The decline was significant (p < 0.001) in 60 days of ASP treated group as compared to the control group. We also observed marked histomorphological changes in ovary and uterus of the ASP treated female mice in duration dependent manner. Female mice treated with ASP showed many histomorphological changes in the ovarian structure including decreased number of growing follicles. Degenerative changes were observed in follicular structure like degenerating oocytes, theca folliculi, corona radiata and zona granulosa. The connective tissue stroma and medulla region also shows some degenerative changes and vacuolation. Aspartame has markedly damaged the reproductive function of the female mice as is evident from the alterations in hormone levels and histomorphological changes in ovary and uterus. The toxic effect of ASP on the reproductive performance of female mice is also manifested by significant (p < 0.05) decrease in pregnancy rate, litter size, litter weight and viability index. This effect may have been caused by uterine and ovarian abnormalities which may have been a direct result of ASP exposure. The ovary is an important target organ of many toxic chemicals and neuroendocrine disruptors [69–72]. We observed that both ovarian and uterine weights were significantly (p < 0.01) decreased in ASP treated animals. These results suggest that ASP may have toxic effects on both ovarian and uterine structures. There are not many studies available on the effect of ASP on gonadotropins and ovarian steroid hormones. A few studies have investigated the effect of ASP on male reproductive system including testosterone hormone and histology of testes, sperm quality, viability and motility [73–75]. A significant decrease in total antioxidant capacity (TAC) and testosterone along with significant increase in malondialdehyde (MDA) was reported following ASP treatment. Aspartame administration decreased number, motility, viability and maturation of sperms along with increased abnormality and DNA damage to sperms [73, 76, 77]. Aspartame administration decreased number, motility, viability and maturation of sperms along with increased abnormality and DNA damage to sperms. Aspartame is reported to have deleterious effects on hypothalamus which produces gonadotropin releasing hormone (GnRH) which goes down the pituitary stalk stimulating the pituitary gland to produce gonadotropins stimulating the testicles and ovaries to produce testosterone [78, 79]. Aspartame is metabolized in the gut releasing the aspartic acid, phenylalanine, methanol, and diketopiperazine. Methanol is converted into formaladehyde and formic acid above 85^o^F and is said to have toxic effects on central nervous system (CNS), gatro-intestinal (GI) tract, liver, kidney and testes [80]. The toxic effects of aspartic acid and methanol on testes are due to the potential of these two components to cross blood-testes barrier and reduce spermatogenesis, reduced tubule size, spermatogenic arrest, and inhibition of steroid biosynthesis in Leydig cells which is an outcome of oxidative stress [81–84].

Chronic administration of ASP (2 mg/g b. w.) induced selective degeneration of all subcelular neurons ultrastructures both in CA1 pyramidal neurons of hippocampus and in ventral-medial area of hypothalamus, which control the activity of pituitary and intense vacuolization like damages [79, 85], loss of the intracytoplasmatic secretory granules in all cellular ultrastructures of the adenohypophysis and therefore alter the homeostasis [86]. Electron-microscopy observations revealed that ASP treatment induced marked alterations in growth hormone (GH) and LH/FSH cells in rats. These alterations were more prominent in either GH or LH/FSH secretory cells, indicating a decline in growth and gonadotropic hormones secretion. Besides, young prepubertal rats appear to be most susceptible to the deleterious effects of ASP [87, 88]. Aspartame induced lesions of the mediobasal hypothalamus are associated with a low release of gonadoliberins and low level of gonadotropic hormones including inhibition of the synthesis and secretion of testosterone. All these changes have an overall effect on the diminution of the reproductive capacity [79]. These observations are in agreement with the finding which shows that administration of excitotoxins (glutamate, aspartic acid, cysteine, and their homologues) to lab rodents through different routes caused neuronal degeneration, associated with axonaldendritic lesions [89–91].

Some studies showed that increased concentrations of ASP metabolites like phenylalanine, aspartic acid, and methanol are responsible for alterations in hormone levels [67, 92, 93]. Increased concentrations of aspartame metabolites were reported in blood following ASP consumption [94]. Excessive phenylalanine interferes with the tyrosine and tryptophan resulting in decreased concentrations of the brain catecholamine, serotonin and dopamine disturbing the balance of neurotransmitters. This in turn leads to neurological, behavioural and hormonal changes [67, 68, 94]. Granulosa cells and follicular membrane cells in the follicle are the target cells of steroid hormones and gonadotropin [95, 96].

We also found that the ovarian and uterine weights were significantly decreased in ASP treated groups. In addition to this, histomorphological changes in both ovary and uterus were also observed in ASP treated groups after different intervals. The changes induced in the ovarian structure after ASP administration characterized by decreased number of growing follicles, degenerative changes were observed in follicular structure i.e. degenerating oocytes, theca layers, corona radiata and zona granulosa. The connective tissue of medulla region also showed some degenerative changes and vacuolization. Besides, ASP also induced histomorphological changes in the uterine structure including atrophic uterine endometrial glands, strained endometrial lining, disruption of the endometrium and the shapes of blood vessels were also altered. Steroid hormones like estradiol (E2) and progesterone (P4) play a very important role in the growth and differentiation of reproductive tissues and maintenance of fertility [97, 98]. Estrogen enhances the sensitivity of granulose cells to FSH and LH, thereby increasing the biosynthesis of progesterone by granulose cells [97, 99]. Estrogen modulates steroidogenesis, promotes granulose cell proliferation and maintains follicular development [100–102]. The release of LH and FSH from the anterior pituitary gland regulates the secretion of reproductive hormones from the ovary [103, 104]. The main function of FSH includes stimulation of ovarian growth and promotion of follicular development. Luteinizing hormone plays an important role in follicular maturation, ovulation, corpus luteum development and is involved in the synthesis of steroid hormones [105–110]. A decreased tendency of females to get pregnant could be due to decreased levels of reproductive hormones. We observed that the endometrium was damaged, and the endometrial glands were atrophied which may be the cause of low fertility rate of ASP treated groups. It is reported that endometrial completeness is critical for the successful implantation of an embryo [111, 112]. Apart from this, a significant decrease in litter size was observed in our study along with decreased body weight of pups in all ASP treated groups. The significant change in gestation period, viability index and weaning index in 90 days ASP treated group. The reduction in body weight of pups is attributed to insufficient availability of substrates including glucose to the foetuses due to possible diminution of substrates in the ASP fed maternal blood [113–116]. It was observed that methanol formed during the ASP metabolism might be responsible for preterm delivery as methanol has been shown to decrease gestational length in primates [65, 117–119].

## Conclusion

In light of the present findings ASP has a correlation with the possibilities of reproductive toxicity. The study conclude that aspartame and its metabolites have the potential to affect female reproductive systems, gestation period and fetal development and pregnancy outcomes. We propose that ASP affects hypothalamic–pituitary–gonadal axis (HPG axis) altering the release of LH and FSH from the anterior pituitary gland and damages the histomorphology of ovary and uterus like follicular maturation, ovulation and corpus luteum development. It is also concluded that uterine endometrium abruption and the atrophy of the uterine glands were the result of ASP intake. ASP decreased the tendency of animals to get pregnant by deminishing the levels of gonandotropins. Small littersize,decreased fetal weight. and extended gestational period supports the conclusion. That aspartame intake should be taken seriously. Aspartame related research investigations are further advised to identify mechanism and pathways affected by ASP consumption and its metabolic breakdown products to understand the molecular mechanism of reproductive alterations and related disorders progression.

## Data Availability

All data generated or analyzed during this study are included in this article [and its supplementary information files].

## Abbreviations

ADI: Acceptable Daily Intake
U.S.: FDA United States Food and Drug Administration
ASs: Artificial Sweeteners
NNSs: Non-nutritive Sweeteners
ASP: Aspartame
IAEC: Institutional Animal Ethical Committee
CCSEA: Committee for Control and Supervision of Experiments on Animals
LH: Luteinizing Hormone
FSH: Follicle Stimulating Hormone
E2: Estrogen (Estradiol)
P4: Progesterone
GSI: Gonadosomatic Index
HPG-axis: Hypothalamic–pituitary–gonadal axis
TAC: total antioxidant capacity
ROS: Reactive oxygen species
MDA: malondialdehyde
LPO: lipid peroxidation
GnRH: gonadotropin releasing hormone
CNS: Central nervous system
GI: tract Gastro-intestinal tract
GH: Growth hormone

## References

[1] Naik AQ, Zafar T, Shrivastava VK (2018). Health implications associated with aspartame consumption: a substantial review. Nutrients.

[2] Ustun B, Reissland N, Covey J, Schaal B, Blissett J (2022). Flavor sensing in Utero and Emerging discriminative behaviors in the human fetus. Psychol Sci.

[3] Mura Paroche M, Caton SJ, Vereijken CMJL, Weenen H, Houston-Price C (2017). How infants and young children learn about food: a systematic review. Front Psychol.

[4] Knüppel A, Shipley MJ, Llewellyn CH, Brunner EJ. Sugar intake from sweet food and beverages, common mental disorder and depression: prospective findings from the Whitehall II study. Sci Rep. 2017;7(1):6287. 10.1038/s41598-017-05649-7. 10.1038/s41598-017-05649-7 PMC5532289 28751637

[5] Freeman CR, Zehra A, Ramirez V, Wiers CE, Volkow ND, Wang GJ (2018). Impact of sugar on the body, brain, and behavior. Front Biosci (Landmark Ed).

[6] Lin X, Xu Y, Pan X (2020). Global, regional, and national burden and trend of diabetes in 195 countries and territories: an analysis from 1990 to 2025. Sci Rep.

[7] Lin X, Li H, Obesity (2021). Epidemiology, pathophysiology, and therapeutics. Front Endocrinol (Lausanne).

[8] Mills KT, Stefanescu A, He J (2020). The global epidemiology of hypertension. Nat Rev Nephrol.

[9] Vaduganathan M, Mensah GA, Turco JV, Fuster V, Roth GA (2022). The Global Burden of Cardiovascular Diseases and Risk: a compass for Future Health. J Am Coll Cardiol.

[10] Veit M, van Asten R, Olie A, Prinz P (2022). The role of dietary sugars, overweight, and obesity in type 2 diabetes mellitus: a narrative review. Eur J Clin Nutr.

[11] Breda J, Jewell J, Keller A (2019). The importance of the World Health Organization Sugar Guidelines for Dental Health and obesity Prevention. Caries Res.

[12] Johnson RK, Appel LJ, Brands M, Howard BV, Lefevre M, Lustig RH, Sacks F, Steffen LM, Wylie-Rosett J, American Heart Association Nutrition Committee of the Council on Nutrition (2009). Physical activity, and metabolism and the Council on Epidemiology and Prevention. Dietary sugars intake and cardiovascular health: a scientific statement from the American Heart Association. Circulation.

[13] Fitch C, Keim KS, Academy of Nutrition and Dietetics (2012). Position of the Academy of Nutrition and Dietetics: use of nutritive and nonnutritive sweeteners [published correction appears in J AcadNutr Diet. 2012;112(8):1279]. J AcadNutr Diet.

[14] Pang MD, Goossens GH, Blaak EE (2021). The impact of Artificial Sweeteners on Body Weight Control and glucose homeostasis. Front Nutr.

[15] Palmnäs MS, Cowan TE, Bomhof MR (2014). Low-dose aspartame consumption differentially affects gut microbiota-host metabolic interactions in the diet-induced obese rat. PLoS ONE.

[16] Adaramoye OA, Akanni OO (2016). Effects of long-term administration of aspartame on biochemical indices, lipid profile and redox status of cellular system of male rats. J Basic Clin PhysiolPharmacol.

[17] Malik VS, Schulze MB, Hu FB (2006). Intake of sugar-sweetened beverages and weight gain: a systematic review. Am J Clin Nutr.

[18] Hu FB (2013). Resolved: there is sufficient scientific evidence that decreasing sugar-sweetened beverage consumption will reduce the prevalence of obesity and obesity-related diseases. Obes Rev.

[19] Seferidi P, Millett C, Laverty AA (2017). Sweetened beverage intake in association to energy and sugar consumption and cardiometabolic markers in children. PediatrObes.

[20] Vos MB, Kaar JL, Welsh JA, Van Horn LV, Feig DI, Anderson C, Patel MJ, Munos C, Krebs J, Xanthakos NF, Johnson SA, American Heart Association Nutrition Committee of the Council on Lifestyle and Cardiometabolic Health; Council on Clinical Cardiology (2017). Council on Cardiovascular Disease in the Young; Council on Cardiovascular and Stroke nursing; Council on Epidemiology and Prevention; Council on Functional Genomics and Translational Biology; and Council on Hypertension. Added Sugars and Cardiovascular Disease Risk in Children: A Scientific Statement from the American Heart Association. Circulation.

[21] Gardner C, Wylie-Rosett J, Gidding SS, Steffen LM, Johnson RK, Reader D, Lichtenstein AH, American Heart Association Nutrition Committee of the Council on Nutrition (2012). Physical activity and metabolism, Council on arteriosclerosis, thrombosis and Vascular Biology, Council on Cardiovascular Disease in the Young, & American Diabetes Association. Nonnutritive sweeteners: current use and health perspectives: a scientific statement from the American Heart Association and the american Diabetes Association. Diabetes Care.

[22] Sørensen LB, Møller P, Flint A, Martens M, Raben A (2003). Effect of sensory perception of foods on appetite and food intake: a review of studies on humans. Int J ObesRelatMetabDisord.

[23] Shankar P, Ahuja S, Sriram K (2013). Non-nutritive sweeteners: review and update. Nutrition.

[24] Miller PE, Perez V (2014). Low-calorie sweeteners and body weight and composition: a meta-analysis of randomized controlled trials and prospective cohort studies. Am J Clin Nutr.

[25] Azad MB, Abou-Setta AM, Chauhan BF (2017). Nonnutritive sweeteners and cardiometabolic health: a systematic review and meta-analysis of randomized controlled trials and prospective cohort studies. CMAJ.

[26] Brown RJ, de Banate MA, Rother KI (2010). Artificial sweeteners: a systematic review of metabolic effects in youth. Int J PediatrObes.

[27] Foreyt J, Kleinman R, Brown RJ, Lindstrom R (2012). The use of low-calorie sweeteners by children: implications for weight management. J Nutr.

[28] Reid AE, Chauhan BF, Rabbani R (2016). Early exposure to nonnutritive sweeteners and long-term Metabolic Health: a systematic review. Pediatrics.

[29] Institute of Medicine. (2007). Nutrition Standards for Foods in Schools: Leading the Way toward Healthier Youth; The National Academies Press: Washington, DC, USA. 2007.

[30] Naik AQ, Zafar T, Shrivastava VK. Physiological impact of the non-nutritive artificial sweetener, aspartame, and the therapeutic potential of aqueous extract of *Phyllanthus niruri*. J Med Food. 2023;26(7):500–10. 10.1089/jmf.2022.K.0136. Epub 2023 May 18. PMID: 37204311. 10.1089/jmf.2022.K.0136 37204311

[31] Sylvetsky AC, Rother KI (2016). Trends in the consumption of low-calorie sweeteners. PhysiolBehav.

[32] Butchko HH, Stargel WW, Comer CP (2002). Aspartame: review of safety. RegulToxicolPharmacol.

[33] Magnuson BA, Burdock GA, Doull J (2007). Aspartame: a safety evaluation based on current use levels, regulations, and toxicological and epidemiological studies. Crit Rev Toxicol.

[34] Swithers SE (2016). Not-so-healthy sugar substitutes?. CurrOpinBehav Sci.

[35] Chaudhari N, Roper SD. The cell biology of taste [published correction appears in J Cell Biol. 2010;191(2):429]. J Cell Biol. 2010;190(3):285–296. 10.1083/jcb.201003144. 10.1083/jcb.201003144 PMC2922655 20696704

[36] Laffitte A, Neiers F, Briand L (2014). Functional roles of the sweet taste receptor in oral and extraoral tissues. CurrOpin Clin NutrMetab Care.

[37] Soffritti M, Belpoggi F, DegliEsposti D, Lambertini L, Tibaldi E, Rigano A (2006). First experimental demonstration of the multipotential carcinogenic effects of aspartame administered in the feed to Sprague-Dawley rats. Environ Health Perspect.

[38] Naik AQ, Shrivastava VK. Effects of short-term consumption of aspartame on some biochemical and hematological parameters in female swiss albino mice. Int J Zool Res 2019;15:21–7. 10.3923/ijzr.2019.21.27.

[39] Anton SD, Martin CK, Han H (2010). Effects of stevia, aspartame, and sucrose on food intake, satiety, and postprandial glucose and insulin levels. Appetite.

[40] Soffritti M, Belpoggi F, Manservigi M (2010). Aspartame administered in feed, beginning prenatally through life span, induces cancers of the liver and lung in male swiss mice. Am J Ind Med.

[41] Halldorsson TI, Strøm M, Petersen SB, Olsen SF (2010). Intake of artificially sweetened soft drinks and risk of preterm delivery: a prospective cohort study in 59,334 danish pregnant women. Am J Clin Nutr.

[42] Abd Elfatah AA, Ghaly IS, Hanafy SM (2012). Cytotoxic effect of aspartame (diet sweet) on the histological and genetic structures of female albino rats and their offspring. Pak J Biol Sci.

[43] Lindseth GN, Coolahan SE, Petros TV, Lindseth PD (2014). Neurobehavioral effects of aspartame consumption. Res Nurs Health.

[44] Abhilash M, Alex M, Mathews VV, Nair RH (2014). Chronic effect of Aspartame on Ionic Homeostasis and Monoamine Neurotransmitters in the rat brain. Int J Toxicol.

[45] Ruiz-Luna AC, Salazar S, Aspajo NJ, Rubio J, Gasco M, Gonzales GF (2005). Lepidium meyenii (Maca) increases litter size in normal adult female mice. Reprod Biol Endocrinol.

[46] Wang N, She Y, Zhu Y (2012). Effects of subchronic aluminum exposure on the reproductive function in female rats. Biol Trace Elem Res.

[47] Chinoy NJ (1993). Essential techniques in Reproductive Physiology and Endocrinology.

[48] Fischer AH, Jacobson KA, Rose J, Zeller R. Hematoxylin and eosin staining of tissue and cell sections. CSH Protoc. 2008;2008. pdb.prot4986. 10.1101/pdb.prot4986 21356829

[49] Rolls BJ (1991). Effects of intense sweeteners on hunger, food intake, and body weight: a review. Am J Clin Nutr.

[50] Rogers PJ, Hogenkamp PS, de Graaf C (2016). Does low-energy sweetener consumption affect energy intake and body weight? A systematic review, including meta-analyses, of the evidence from human and animal studies. Int J Obes (Lond).

[51] Hall WL, Millward DJ, Rogers PJ, Morgan LM (2003). Physiological mechanisms mediating aspartame-induced satiety. PhysiolBehav.

[52] Kissileff HR, Gordon RJ, Thornton JC (2019). Combined effects of cholecystokinin-8 and gastric distension on food intake in humans. Am J PhysiolRegulIntegr Comp Physiol.

[53] Fitzgerald PCE, Manoliu B, Herbillon B, Steinert RE, Horowitz M, Feinle-Bisset C (2020). Effects of L-Phenylalanine on Energy Intake and Glycaemia-Impacts on Appetite perceptions, gastrointestinal hormones and gastric emptying in healthy males. Nutrients.

[54] Hunty ADL, Gibson S, Ashwell M (2006). A review of the effectiveness of aspartame in helping with weight control. Nutr Bull.

[55] Swithers SE, Davidson TL (2008). A role for sweet taste: calorie predictive relations in energy regulation by rats. Behavneurosci.

[56] Ragi ME, El-Haber R, El-Masri F, Obeid OA (2022). The effect of aspartame and sucralose intake on body weight measures and blood metabolites: role of their form (solid and/or liquid) of ingestion. Br J Nutr.

[57] Beck B, Burlet A, Max JP, Stricker-Krongrad A (2002). Effects of long-term ingestion of aspartame on hypothalamic neuropeptide Y, plasma leptin and body weight gain and composition. PhysiolBehav.

[58] Sousa-Ferreira L, Garrido M, Nascimento-Ferreira I (2011). Moderate long-term modulation of neuropeptide Y in hypothalamic arcuate nucleus induces energy balance alterations in adult rats. PLoS ONE.

[59] Shin BC, Dai Y, Thamotharan M, Gibson LC, Devaskar SU (2012). Pre- and postnatal calorie restriction perturbs early hypothalamic neuropeptide and energy balance. J Neurosci Res.

[60] Zhang QJ, Yang CC, Zhang SY, Zhang LH, Li J (2016). Alteration of NPY in hypothalamus and its correlation with leptin and ghrelin during the development of T2DM in a rat model. Springerplus.

[61] Su Y, Foppen E, Fliers E, Kalsbeek A (2016). Effects of Intracerebroventricular Administration of Neuropeptide Y on metabolic gene expression and energy metabolism in male rats. Endocrinology.

[62] Parthasarathy NJ, Kumar RS, Manikandan S, Devi RS (2006). Methanol-induced oxidative stress in rat lymphoid organs. J Occup Health.

[63] Saoudi M, Ben Hsouna A, Trigui M, Jamoussi K, Jaoua S, El Feki A (2012). Differential oxidative stress responses to methanol in intraperitoneally exposed rats: ameliorative effects of Opuntia vulgaris fruit extract. Toxicol Ind Health.

[64] Parthasarathy NJ, Srikumar R, Manikandan S, Narayanan GS, Devi RS (2007). Effect of methanol intoxication on specific immune functions of albino rats. Cell Biol Toxicol.

[65] Choudhary AK, Sheela Devi R (2015). Longer period of oral administration of aspartame on cytokine response in Wistar albino rats. Endocrinol Nutr.

[66] Nakao H, Umebayashi C, Nakata M (2003). Formaldehyde-induced shrinkage of rat thymocytes. J Pharmacol Sci.

[67] Ashok I, Sheeladevi R, Wankhar D (2015). Acute effect of aspartame-induced oxidative stress in Wistar albino rat brain. J Biomed Res.

[68] Ashok I, Wankhar D, Wankhar W, Sheeladevi R (2015). Neurobehavioral changes and activation of neurodegenerative apoptosis on long-term consumption of aspartame in the rat brain. J NutrIntermedMetabol.

[69] Patel S, Zhou C, Rattan S, Flaws JA (2015). Effects of endocrine-disrupting chemicals on the Ovary. Biol Reprod.

[70] Sifakis S, Androutsopoulos VP, Tsatsakis AM, Spandidos DA (2017). Human exposure to endocrine disrupting chemicals: effects on the male and female reproductive systems. Environ Toxicol Pharmacol.

[71] Mourikes VE, Flaws JA (2021). Reproductive, Toxicology: Effects of chemical mixtures on the ovary. Reproduction.

[72] Ghosh A, Tripathy A, Ghosh D (2022). Impact of endocrine disrupting chemicals (EDCs) on Reproductive Health of Human. Proc Zool Soc.

[73] Anbara H, Sheibani M, Razi M (2020). Long-term effect of Aspartame on Male Reproductive System: evidence for testicular histomorphometrics, Hsp70-2 protein expression and biochemical status. Int J FertilAndSteril.

[74] Anbara H, Sheibani MT, Razi M, Kian M (2021). Insight into the mechanism of aspartame-induced toxicity in male reproductive system following long-term consumption in mice model. Environ Toxicol.

[75] Kearns ML, MacAindriu F, Reynolds CM (2022). The impact of non-caloric sweeteners on male fertility: a systematic review and narrative synthesis in Rodent Models. Front Nutr.

[76] Hannon PR, Flaws JA (2015). The effects of phthalates on the ovary. Front Endocrinol (Lausanne).

[77] Sheibani MT, Anbara H, Morovvati H, Razi M, SalarAmoli J (2019). Effect of Long term-administration of Aspartame on sperm quality, testosterone and oxidant parameters in mice. J Ilam Uni Med Sci.

[78] USEPA: Guidelines for Reproductive Toxicity Risk Assessment (1996). Risk Assessment Forum U.S. Environmental Protection Agency Washington. DC Fed Register.

[79] Puica C, Craciun C, Rusu M, Cristescu M, Borsa M, Roman I (2009). Ultrastructural aspects concerning the hypothalamus-pituitary complex reactivity following chronic administration of aspartame in juvenile rats. Studia Universitatis “VasileGoldiş” SeriaŞtiințeleVieţii.

[80] Pohanish RP. Sittig’s handbook of toxic and hazardous chemicals and carcinogens. Seventh ed. Elsevier, William Andrew Publishing; 2017. pp. 3224–31.

[81] Henkel R, Kierspel E, Stalf T (2005). Effect of reactive oxygen species produced by spermatozoa and leukocytes on sperm functions in non-leukocytospermic patients. FertilSteril.

[82] Ruwanpura SM, McLachlan RI, Stanton PG, Loveland KL, Meachem SJ (2008). Pathways involved in testicular germ cell apoptosis in immature rats after FSH suppression. J Endocrinol.

[83] Del Olmo E, Bisbal A, García-Álvarez O (2015). Free-radical production after post-thaw incubation of ram spermatozoa is related to decrease in vivo fertility. ReprodFertil Dev.

[84] Seifried RM, Harrison E, Seifried HE. Antioxidants in Health and Disease, In: Coulston AM, Boushey CJ, Ferruzzi MG, Delahanty LM, editors. Nutrition in the Prevention and Treatment of Disease (Fourth Edition). 2017. USA: Academic Press; 2007. p. 321–346.

[85] Collison KS, Inglis A, Shibin S (2018). Effect of developmental NMDAR antagonism with CGP 39551 on aspartame-induced hypothalamic and adrenal gene expression. PLoS ONE.

[86] Park S, Sethi S, Bouret SG (2019). Non-nutritive sweeteners induce hypothalamic ER stress causing abnormal Axon Outgrowth. Front Endocrinol (Lausanne).

[87] Azeez OH (2022). Evaluation of some male and female rats’ Reproductive Hormones following administration of Aspartame with or without vitamin C or E. Iraqi J Vet Med.

[88] Chen YC, Yeh YC, Lin YF (2022). Aspartame Consumption, mitochondrial Disorder-Induced impaired ovarian function, and infertility risk. Int J Mol Sci.

[89] Lau K, McLean WG, Williams DP, Howard CV (2006). Synergistic interactions between commonly used food additives in a developmental neurotoxicity test. Toxicol Sci.

[90] Mattson MP. (2007). Excitotoxins, In: Fink G. editor. Encyclopedia of Stress. Amsterdam: Academic Press; 2007. p. 975–982.

[91] Dong XX, Wang Y, Qin ZH (2009). Molecular mechanisms of excitotoxicity and their relevance to pathogenesis of neurodegenerative diseases. Acta Pharmacol Sin.

[92] Choudhary AK, Devi RS (2014). Imbalance of the oxidant - antioxidant status by aspartame in the organs of immune system of Wistar albino rats. Afr J Pharm Pharmacol.

[93] Choudhary AK, Devi RS (2014). Serum biochemical responses under oxidative stress of aspartame in Wistar albino rats. Asian Pac J Trop Dis.

[94] Humphries P, Pretorius E, Naudé H (2008). Direct and indirect cellular effects of aspartame on the brain. Eur J Clin Nutr.

[95] Drummond AE. The role of steroids in follicular growth. Reprod Biol Endocrinol. 2006;4:16. 10.1186/1477-7827-4-16. 10.1186/1477-7827-4-16 PMC1459164 16603089

[96] Holesh JE, Bass AN, Lord M, Physiology. Ovulation. In: *StatPearls*. Treasure Island (FL): StatPearls Publishing; May 8, 2022. 28723025

[97] da Silva Faria T, de Bittencourt Brasil F, Sampaio FJ, da Fonte Ramos C (2010). Effects of maternal under-nutrition during lactation on estrogen and androgen receptor expressions in rat ovary at puberty. Nutrition.

[98] Sloboda DM, Hickey M, Hart R (2011). Reproduction in females: the role of the early life environment. Hum Reprod Update.

[99] Taraborrelli S (2015). Physiology, production and action of progesterone. Acta ObstetGynecol Scand.

[100] Shimizu T, Hirai Y, Miyamoto A (2013). Expression of cyclins and cyclin-dependent kinase inhibitors in granulosa cells from bovine ovary. ReprodDomest Anim.

[101] Chan KA, Tsoulis MW, Sloboda DM (2015). Early-life nutritional effects on the female reproductive system. J Endocrinol.

[102] Chauvin S, Cohen-Tannoudji J, Guigon CJ (2022). Estradiol Signaling at the heart of Folliculogenesis: its potential deregulation in human ovarian pathologies. Int J Mol Sci.

[103] Allan CM, Wang Y, Jimenez M (2006). Follicle-stimulating hormone increases primordial follicle reserve in mature female hypogonadal mice. J Endocrinol.

[104] François CM, Petit F, Giton F et al. A novel action of follicle-stimulating hormone in the ovary promotes estradiol production without inducing excessive follicular growth before puberty. Sci Rep. 2017;7:46222. 10.1038/srep46222. 10.1038/srep46222 PMC5387682 28397811

[105] Hunter MG, Robinson RS, Mann GE, Webb R (2004). Endocrine and paracrine control of follicular development and ovulation rate in farm species. Anim Reprod Sci.

[106] Stocco C, Telleria C, Gibori G (2007). The molecular control of corpus luteum formation, function, and regression. Endocr Rev.

[107] Juengel JL, Smith PR, Quirke LD, French MC, Edwards SJ (2018). The local regulation of folliculogenesis by members of the transforming growth factor superfamily and its relevance for advanced breeding programmes. Anim Reprod.

[108] Juengel JL, Cushman RA, Dupont J (2021). The ovarian follicle of ruminants: the path from conceptus to adult. Reprod Fertil Dev.

[109] Zerani M, Polisca A, Boiti C, Maranesi M (2021). Current knowledge on the multifactorial regulation of Corpora Lutea Lifespan: the rabbit model. Anim (Basel).

[110] Nedresky D, Singh G, Physiology. Luteinizing hormone. In: *StatPearls*. Treasure Island (FL): StatPearls Publishing; September 26, 2022. 30969514

[111] Zhou Y, Zhang H, He J (2013). Effects of sodium fluoride on reproductive function in female rats. Food Chem Toxicol.

[112] Dong S, Yang Y, He B (2023). Effect of Sodium Fluoride on Reproductive function through regulating Reproductive hormone level and circulating SIRT1 in female rats. Biol Trace Elem Res.

[113] Leme LF, Azoubel R (2006). Effects of aspartame on the exocrine pancreas of rat fetuses. Int J Morphol.

[114] Martins MRI, Azoubel R (2007). Effects of aspartame on fetal kidney: a morphometry and stereological study. Int J Morphol.

[115] Portela GS, Azoubel R, Batigalia F (2007). Effects of aspartame on maternal-fetal and placental weights, length of umbilical cord and fetal liver: a kariometric experimental study. Int J Morphol.

[116] Goran MI, Plows JF, Ventura EE (2019). Effects of consuming sugars and alternative sweeteners during pregnancy on maternal and child health: evidence for a secondhand sugar effect. Proc Nutr Soc.

[117] Trocho C, Pardo R, Rafecas I (1998). Formaldehyde derived from dietary aspartame binds to tissue components in vivo. Life Sci.

[118] Burbacher TM, Grant KS, Shen DD (2004). Chronic maternal methanol inhalation in nonhuman primates (*Macaca fascicularis*): reproductive performance and birth outcome. NeurotoxicolTeratol.

[119] Alkafafy Mel -S, Ibrahim ZS, Ahmed MM, El-Shazly SA (2015). Impact of aspartame and saccharin on the rat liver: biochemical, molecular, and histological approach. Int J ImmunopatholPharmacol.

